# Pavlovian-Instrumental Interaction in ‘Observing Behavior’

**DOI:** 10.1371/journal.pcbi.1000903

**Published:** 2010-09-09

**Authors:** Ulrik R. Beierholm, Peter Dayan

**Affiliations:** Gatsby Computational Neuroscience Unit, University College London, London, United Kingdom; John Radcliffe Hospital, United Kingdom

## Abstract

Subjects typically choose to be presented with stimuli that predict the existence of future reinforcements. This so-called ‘observing behavior’ is evident in many species under various experimental conditions, including if the choice is expensive, or if there is nothing that subjects can do to improve their lot with the information gained. A recent study showed that the activities of putative midbrain dopamine neurons reflect this preference for observation in a way that appears to challenge the common prediction-error interpretation of these neurons. In this paper, we provide an alternative account according to which observing behavior arises from a small, possibly Pavlovian, bias associated with the operation of working memory.

## Author Summary

The theory of Reinforcement Learning (RL) has been influential in explaining basic learning and behavior in humans and other animals, and in accounting for key features of the activity of dopamine neurons. However, perhaps due to this very success, paradigms that challenge RL are at a premium. One case concerns so-called ‘*observing behavior*’, in which, at least in some versions, animals elect to observe cues that are predictive of future rewarding outcomes, although the observations themselves have no direct behavioral relevance. In a recent experiment on observing, the activity of monkey dopaminergic neurons was also found to be incompatible with classic RL. However, as is often the case, this was a task that allowed for potential interactions from a secondary behavioral system in which responses are directly triggered by values. In this paper we show that a model incorporating a next order of refinement associated with such Pavlovian interactions can explain this type of observing behavior.

## Introduction

Animal behavior all too rarely follows the precepts of simple theories such as normatively optimal choice. Prominent examples of this arise in the florid fancies of Breland & Breland's animal actors [1], or in the complexities of negative automaintenance or omission schedules [2]–[4]. Such failures and irrationalities have been important sources of theory revision and refinement, for instance leading to suggestions about the competition and cooperation of multiple systems of control [5]–[7], some instrumental and adaptive; others Pavlovian and hard-wired.

In this paper, we study one apparent departure from optimality, namely a type of ‘observing behavior’ [8], [9], which has been the subject of a recent important electrophysiological study [10]. In brief, subjects are programmed to receive either a large or small reward, with its size being determined stochastically. When faced with the choice of finding out (by being presented with a suitably distinctive cue) sooner rather than later which of the two rewards they will ultimately receive, subjects prefer to know sooner. A lack of indifference despite the equality of the outcomes has been found to be widely true even if the knowledge cannot influence the outcome, and, at least in other experiments, even if this choice is expensive [8], [9], [11]–[13]. In economics, the same anomaly is referred to in terms of “temporal resolution of uncertainty” [14], explained by such notions as savoring [15]–[17], with subjects enjoying the anticipation of good things to come.

The correct interpretation of this form of observing behavior has been the subject of substantial debate (see, e.g. [9]). Superficially attractive theories, such as a desire to gain Shannon information [18] have been dealt fatal blows, for instance with animals preferring to observe *more* even when the number of bits they receive by doing so is *less* (e.g., as the probability of getting the large reward becomes smaller than 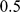, [12]).

A recent study on observing behavior in macaques [10] has offered a new perspective on the problem. These authors recorded from putative dopamine neurons in the midbrain whilst monkeys chose to observe. According to a common theory, these neurons report a temporal difference error in predictions of future reward [19], [20] as in reinforcement learning accounts of optimal instrumental choice [21]. Bromberg-Martin and Hikosaka [10] showed that: (a) the macaques did observe; and furthermore (b) the activity of dopamine neurons was associated with the choice they make. However, although the behavior and activity are mutually consistent, observing behavior offers no instrumental benefit and therefore it should also not be associated with any prediction errors. Bromberg-Martin and Hikosaka suggested that this means that the dopamine cells are reporting on some aspects of the benefit of information gathering in addition to aspects of reward.

In this paper, we examine the extent to which this form of observing behavior can be explained by temporal difference learning, coupled with the same mechanism that provides an account of a wide range of departures from normative choice, namely a Pavlovian influence over instrumental actions [4]. In particular, we assume that subjects only make associative predictions when they are appropriately engaged in the task. If the level of this engagement is influenced by the size of the predictions (the putatively Pavlovian effect), then stimuli predicting certain or deterministic large future rewards (one outcome of an observing choice) will lead to more engagement than stimuli that leave uncertain the magnitude of the future rewards. This idea can be seen as a realization of the suggestion made by Dinsmoor [9] that the predictions of future reward associated with stimuli influence the attention paid to them. We show that occasional failures of engagement, modeled as a breakdown in the working memory for the representational state, can lead directly to both the preference for observing and the apparently anomalous dopamine activity, without need for any reference to ‘information’. We also examine the various factors that control the strength of observing in this model.

## Results

**Figure 1:**
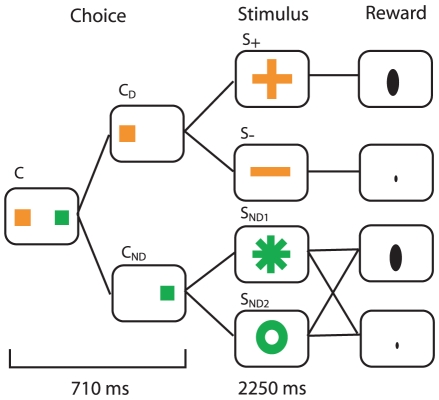
Experimental setup for a free-choice trial, similar to Bromberg-Martin and Hikosaka [10]. The monkey performs its choice (C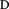 or C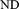) according to color, and the discriminating/random stimulus is presented. At the end of the trial either a large (1 ml) or tiny (0.04 ml) amount of water is delivered.

Bromberg-Martin and Hikosaka's experiment (see Methods and Figure 1) involved the most precise conditions for establishing observing behavior. On each trial, thirsty subjects had a 50% chance of receiving a small or large volume of water directly into their mouths. There were three sorts of trials: forced-information, forced-random and free choice. On forced-information trials, the subjects were presented with a single target (C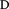; just an orange square in the figure) and, after looking at it, would receive one of two cues (S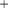; an orange ‘+’, or S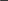; an orange ‘
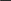
’) according to the volume they were to receive in a couple of seconds. On forced-random trials, looking at the single target (C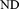; green square) led again to one of two cues (S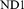; green ‘*’, or S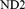; green ‘o’). However, either of these could be followed by either small or large rewards; and thus they provided no discriminative information about the forthcoming reward. Finally, on free choice trials, both orange and green targets were provided, and the subjects could choose whether to receive the discriminative (orange) or non-discriminative (green) cues.

**Figure 2:**
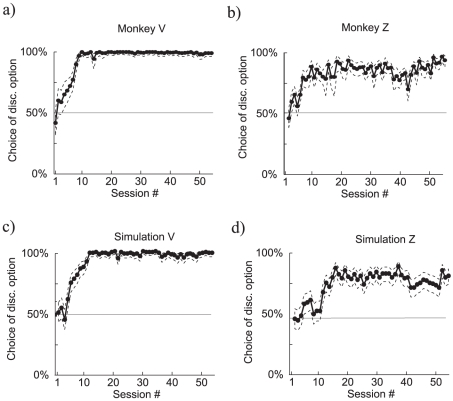
Comparing observing in monkeys and the model. a–b) Observing in two monkeys performing the task, from Bromberg-Martin and Hikosaka [10]. The dotted lines correspond to the Clopper-Pearson 95 percent confidence interval. c–d) Two examples of observing produced by the model. The parameters for the two plots differ only by the parameter 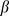, the inverse temperature in the softmax. Each session is 480 trials in the simulations (160 choice trials).

Figures 2a;b show primary behavioral results from the study for two subjects – both gradually expressed a bias towards the discriminative (orange) option in the free-choice trials. As Bromberg-Martin and Hikosaka stressed, under a standard associative learning or temporal difference scheme, there is no difference between the expected reward for the discriminating and non-discriminating option, and so no reason to expect this strong and enduring preference.

We built a model of this which, with one critical exception that we discuss below, involves a standard temporal difference learning algorithm [21], [22]. Forced-choice and free-choice trials permit learning about the future expected rewards associated with the various targets and stimuli, training the values of the states. Then, on free-choice trials, the selection depends on the relative values, via a softmax function (see methods). Figure 2c;d shows the results from simulations of our model, with parameters chosen to match Bromberg-Martin and Hikosaka's two subjects. The model closely matches qualitative features of the monkeys' performances.

In standard models such as this, in which there is a delay between the presentation of cues and the rewards that they predict, an assumption has to be made about the way that the subjects maintain knowledge about their state in the task, and indeed keep time. Many different possibilities have been explored, from delay lines to complex patterns of activity evolving in dynamical recurrent networks (e.g., [23]–[28]). All of these amount to forms of working memory – and so present the minimal requirement that the subjects continue to be engaged in the task throughout the delay in sufficiently intense a manner as to maintain this ongoing memory. Thus the critical exception to conventional temporal difference learning in our model is to assume that this maintained engagement is influenced by the current predicted value. That is, if the value is high, then engagement is readily maintained; if the value is low, then engagement can be weakened or lost.

Losing engagement is detrimental to the subject in the context of the present task; by analogy with a similarly detrimental effect in negative automaintenance, we consider it a form of Pavlovian misbehavior [4]. Pavlovian responses are typically elicited in an automatic manner based on appetitive or aversive predictions, and can exert benign or malign influences over the achievement of subjects' apparent goals. Normally, such responses are overt behaviors; here, along with several recent studies [29], [30], we consider internal responses, associated with the operation of working memory. Mechanistically, these could come, for instance, from the influence dopamine itself exerts on the processes concerned [31].

**Figure 3:**
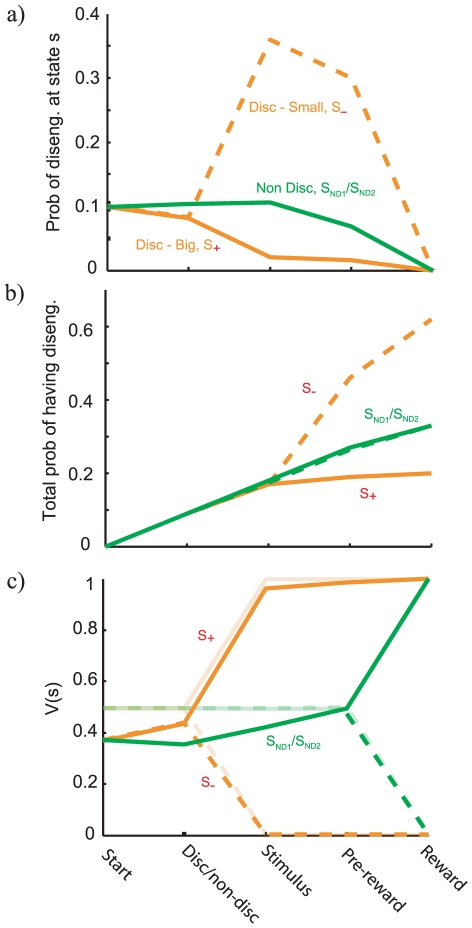
The mechanics of the model. a) The probability of disengagement at different timepoints for the task in Figure 1 (conditional on having not disengaged at prior timesteps). Similar color convention as in Figure 1. Orange traces are for discriminative trials; green for non-discriminative ones; solid lines for the larger reward or one of the two non-discriminative cues; dashed lines for the smaller reward. b) The total probability of having disengaged by the time of reaching state 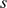. c) The expected reward, V, at different timepoints for the TD model with disengagement. For comparison, the expected reward for a traditional TD model without disengagement is shown in transparent colors. Notice that although the chance of disengagement is high for S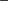, it has little effect due to the already low value of this state. By contrast, the moderate engagement for S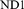 and S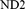 has a larger effect due to their higher associated value.

In the model, we consider engagement to be lost completely on some trials as a stochastic function of the evolving predicted value. Such losses have the effect of decreasing the subjective value of cues and states associated with lower values below their objective worth; in particular exerting a negative bias on the non-discriminative cues (S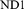; S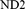) compared with the discriminative cue associated with the large reward (S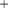), which will more rarely experience such losses. Figure 3 shows the effective probability of disengagement at different timepoints as well as showing the effect this has on the expected reward. Disengagement associated with S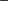 is benign, since the outcome on those trials is modelled as being close to 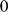 in any case. Altogether, this creates a bias towards choosing the discriminative option on free-choice trials, as is evident in Figure 2c;d.

The difference between the parameters for Figures 2c;d is in the parameter 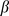 governing the strength of the competition in the softmax (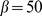 and 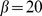 for Figure 2c;d respectively). Monkey V's results are consistent with a larger value of 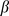 than monkey Z; smaller 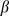 leads to more stochasticity and a lower overall degree of preference. The asymptotic preference for observing is monotonic in 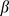.

**Figure 4:**
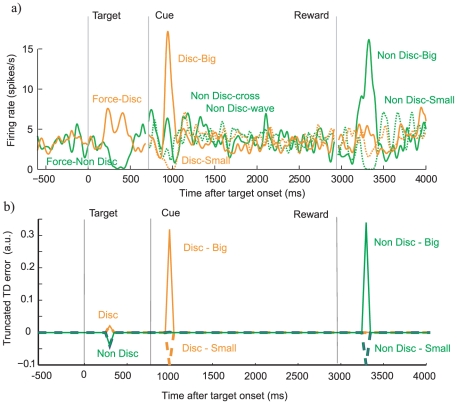
Comparison of neuronal firing and the modeled TD signal. a) An example of the firing rate of a single dopamine neuron during forced trials, based on data from [10]. The various trial types are marked on the plot; briefly orange traces are for discriminative trials; green for non-discriminative ones; solid lines for the larger reward, when known (or one of the two non-discriminative cues); dashed lines for the smaller reward (or the other non-discriminative cue). b) The modeled average TD signal at different time points in a trial using the same conventions as in (a). In order to facilitate the visual comparison of model and data in this figure, we truncated the negative part of the modeled TD signal at 25% of the maximal positive response of the neuron.

Bromberg-Martin and Hikosaka [10] also recorded the activity of putative midbrain dopaminergic cells during the performance of the task. Figure 4a shows the activity of an example neuron in the various conditions. The population response is similar (Figure 4 of [10]) albeit, as has often been seen, with an initial brief activation to the forced choice non-discriminative case, likely because of generalization [32]. Firing at the time of the discriminative or non-discriminative cues (marked ‘cue’) and the delivery or non-delivery of reward (‘reward’) is just as expected from the standard interpretation of these neurons, i.e., that they report the temporal difference prediction error in the delivery of future reward [19], [20].

However, it is their activity at the time of the targets indicating the forced-informative or forced-random trials (marked ‘target’) that is revealing about observing. The target indicating a forced-informative trial was associated with a small but significant phasic increase in activity; whereas that indicating the random cues was followed by a small decrease in the firing rate. Under the temporal difference interpretation of the neurons, this is consistent with the preference exhibited by the monkeys, but not with the objective value of the options.

Figure 4b shows modelled dopamine activity in the variable engagement temporal difference model (here, negative prediction errors have been compressed compared with positive ones, see methods; [33], [34]). This shows exactly the same pattern shown in the monkey data. Note that, once the subject has learned the associations and learned the preference for choosing the discriminative option in the free choice trials, these trials will overall be more frequent than the forced-random trials, and so the negative prediction error associated with the latter will be larger than the positive prediction error associated with the former.

**Figure 5:**
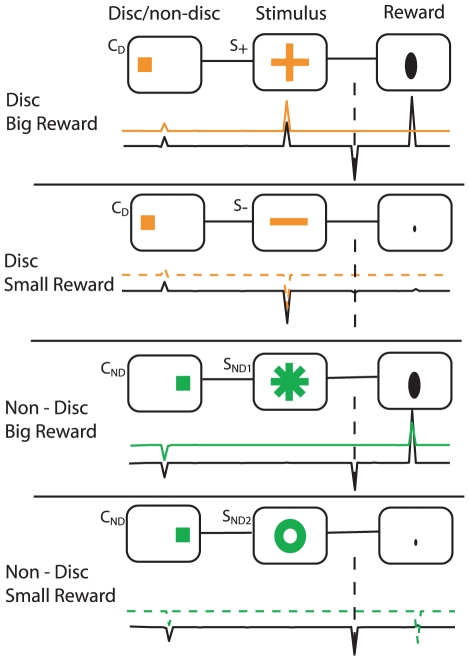
An illustrative example of the modeled temporal difference signal for each of the four conditions. The coloured line indicates the regular temporal difference term, with the following color convention: orange represents a discriminating choice, green is the non-discriminating option, while the complete line is for a rewarding trial, the dotted line for a non rewarding trial. The vertically off-set black line represents the temporal difference signal for a failure after the time of the revealing (indicated by the black dotted line) of the stimulus due to Pavlovian dis-engagement. Notice that the dis-engagement is an unlikely event that relatively rarely elicits a dip in the TD signal, whereas, e.g., the delivery of an unexpected reward elicits the typically robust response.

Figure 5 decomposes the modelled responses in the cases that there is successful and failed engagement between cues and reward or non-reward. The most significant effect of the complete failure to engage given an non-discriminative cue, is that if the large reward is provided, then there is a greater response than expected from a 50% prediction. The possibility of using this to test the theory is discussed below.

**Figure 6:**
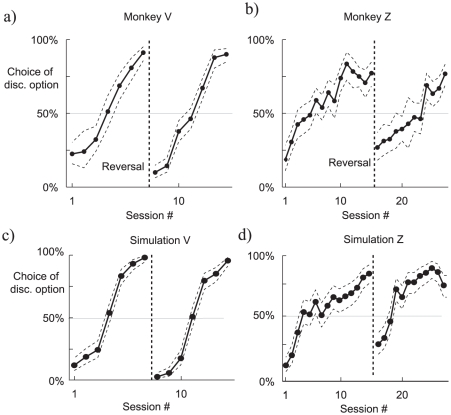
Comparison of observing in monkeys and the model for a delayed task. a–b) The biases of two monkeys performing a version of the observing task in which they were given the choice of receiving immediate or delayed discriminating stimuli, from Bromberg-Martin and Hikosaka. The colors of the choices switched in the session number indicated in the graph. The dotted lines correspond to the Clopper-Pearson 95 percent confidence interval. c–d) Two examples of biasing in switching, similar to Bromberg-Martin and Hikosaka. The parameters for the two plots differ only by the 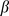 in the softmax (same values as in Figure 2c;d).

In a version of the task that involved choice between immediate or delayed information about upcoming rewards, Bromberg-Martin and Hikosaka [10] further showed that switching the colors of the cues without warning led to a slow reversal of the observing choice (Figure 6a;b). Figure 6c;d shows the same for the model using identical softmax parameters to those in Figure 2c;d. The switch in preference evolves at a similarly glacial pace.

**Figure 7:**
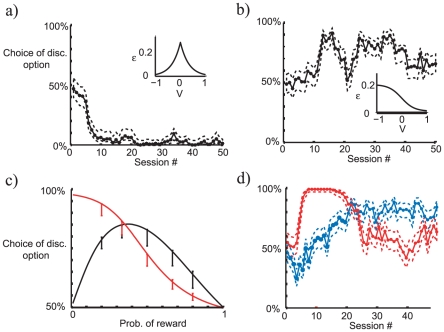
Effect of varying parameters in the model. a–b) For aversive stimuli (punishment) the shape of the memory retention as a function of the expected value has a large effect on the bias towards observing. A symmetric function (a) leads to less observing, an asymmetric function (b) leads to more observing. The dotted lines indicates the Clopper-Pearson 95 percent confidence interval. c) Given appetitive stimuli, the rate of reward 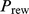 can have different effects on the tendency to choose the discriminating option, based on the version of softmax used. The iterative solution to the self-consistency requirement (6) using the softmax from Eq. 5 (black) is plotted, as well as the iterative solution for choices based on the logarithm of the learned value (red). Mean choice bias for Monte carlo simulations (with STD) are overlaid for 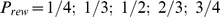. d) Given initial starting conditions far from the correct values, initial learning can lead to too strong or weak effects (single runs; initial values of all states are 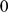 and 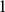 and 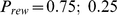 for the blue and red curves respectively).

Various other features of observing can be examined through the medium of the model. Figure 7a;b show the consequence of the reinforcing outcome being aversive (e.g., an electric shock) rather than appetitive. One key question in this case is whether failure to engage is controlled more by salience or valence. Figure 7a shows the former case, for which a prediction of a large punishment also protects engagement (symmetrically with reward; inset plot). In this case, subjects prefer the random to the discriminative cues, since disengagement leads to subjective preference. Such preference for random cues might also come from adding a fixed value to all the potential rewards, thus allowing the moderately large disengagement in S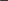 to have a subtractive value on its expected values (Bromberg-Martin, personal communication, 2010). However such an effect would likely be small.

Figure 7b shows the case in which valence (from appetitive to aversive) determines disengagement, with predictions of punishments leading to more failures of engagement than small rewards. This again supports observing behavior. Unfortunately, experimental tests of the case involving punishment [35] have not enjoyed the precision of the paradigm adopted by Bromberg-Martin and Hikosaka, leaving open the question as to which of these patterns arises.

Another important experimental manipulation has been to vary the probability 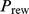 of the larger versus the smaller reward. As 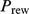 decreases from 1 towards 0.5 there is an increase in the observing bias (i.e., a greater tendency to choose the discriminative option). Below this, the nature of the bias depends on the assumption about how the choices are generated. A choice rule that depends on the difference in expected values (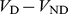) leads to a bias that ultimately decreases towards 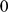 as these values themselves decrease towards 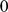. However, the bias is asymmetric about 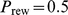 (black curve in Figure 7c). If, instead, the choices are based on the ratio of the values (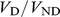), the choice bias can continue to increase as 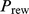 approaches 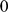 (red curve). Just such an increase in observing was shown by Roper and Zentall [12] as reward schedules thinned. While some studies have also manipulated the size of the reward [36]–[38], our model does not make any direct predictions about this. It is possible that adaptation would scale the response to the overall sizes of available rewards (as indeed found for phasic dopamine activity in [39]), and the metrics of this would have to be known in order to make predictions about disengagement.

One extra factor that is important for analysing behavior is that the biases inherent in disengagement are small and develop over a long time-scale, consistent with the stately progress evident in Figure 2. However, this means that the initial course of learning can be subject to significant influence from the initial values ascribed to the different options, leading to biases that are incommensurate with the final, long term, state. Figure 7d shows an example. For the blue curve, the initial values of all states are low (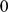), but the probability of a reward is high (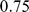); for the red curve, the initial values are high (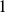), but the probability of a reward is low (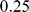). In the former case, there is substantial initial over-observation; in the latter, initial under-observation.

## Discussion

We have provided an account of ‘observing behavior’ that shows how it can arise from a small Pavlovian bias over instrumental behavior associated with disengagement from a task, rather than any aspect of information seeking. Pavlovian biases are rife in decision-making; and accommodating them does not necessitate any further change to the standard underlying theory of the activity of dopaminergic neurons that has not already been suggested to accommodate other data. What we have done here is specify the shape of such an interaction based on disengagement in the task. We intended specifically to capture [10] experiment on macaques. However our results do touch upon other, but emphatically not all, instances of observing in the literature.

Experiments such as [10] into observing are designed to maximize the effects of what is a relatively small anomaly in decision making (compared, for instance, with the more extreme misbehavior evident in negative automaintenance [2] or the schedule task [40]). Indeed, in this case, the subjects did not have to pay a penalty for observing. Thus, under standard decision-making conditions, we may expect the net effect of disengagement to be modest, leaving near-optimal behavior within the scope of the model.

Dinsmoor [9] suggested an account of the phenomenon based on his observation of ‘selective observing’, i.e., that the subjects would preferentially focus on stimuli associated with higher probabilities of reward. This idea met some resistance (some of which is contained in the commentary to [9]), partly based on experimental tests in which the subjects were not able to avoid the low value predictive cues. Our account can be seen as a form of selective observing, but involving internal actions associated with the allocation of engagement and attention, rather than external actions involving preferential looking. It might seem that these accounts are close to Mackintosh's [41] suggestion that attention is preferentially paid to stimuli that are strong predictors of affectively important outcomes. However, in Mackintosh's account, attention particularly influences the speed of learning (the associability of the stimulus) rather than the fact of it (at least in the absence of competing predictors), and so would not have the asymptotic effect that is apparent in the experiments we have discussed.

Another interesting account of observing is Daly and Daly's DMOD [42], which learns predictions associated with frustration (when reward is expected, but does not arrive), and courage (when reward is actually delivered during a state of frustration). These extra predictions warp the net expected values associated with the different cases in observing, favoring observing responses. The theory underlying DMOD is the original Rescorla-Wagner [43] version of the delta rule [44], whose substantial modification by Sutton and Barto [45] to account for secondary conditioning led to the original prediction error treatment of the activity of dopamine neurons in appetitive conditioning [19]. It would be necessary to extend DMOD in a similar way, and to make an assumption about which of its three prediction errors (or other quantities) are reflected in the activity of dopamine neurons, in order to determine its match to the neurophysiological data. The failure of TD models to capture behavioral aspects of frustration is, however, notable.

To some tastes, the most theoretically appealing accounts of observing start from the notion that animals seek to acquire information about the world [46]. However, formal informational theories have difficulty with the results of reducing the probability of reward (Figure 7c; [12]), which reduce the uncertainty and the information gained, but increase observing. More informal theories, such as that suggested by [10] require more precise specification to be tested against accounts such as the one here. The sloth of initial learning and reversal apparent in Figure 6 (taking 1200–2400 choice trials, 3000–7000 trials overall) might be considered suggestive evidence against an informational account, since it implies at the very least a nugatory value for the information.

In terms of our account, there are various routes by which predicted values could influence persistent engagement. Failure to engage can be seen as the same sort of malign Pavlovian influence over behavior that is implicated in the poor performance of monkeys in tasks in which they know themselves to be several steps away from reward [40], [47]. In that paradigm, it is an explicitly informative cue that the reward is disappointingly far away that leads to disengagement; this parallels the disappointment associated with the non-discriminative cue in observing. The most obvious mechanism associated with engagement is the influence of dopamine itself over working memory [31]; however, whether this is the phasic dopamine signal associated with prediction errors for reward [19] or a more tonic dopamine signal associated with a longer term average reward rate [48], [49] is not clear. Alternatively, some theories suggest that working memory is controlled by a gating process [29], [30] associated with the basal ganglia, treating internally- and externally directed action in a uniform manner. Dopamine certainly influences the vigor associated with external actions [48]–[50]; it is therefore reasonable to assume that it might also influence internal engagement.

We specialized our description of the model to the particulars of the experiment conducted by Bromberg-Martin and Hikosaka [10]. The most important question for other cases concerns the conditions under which re-engagement occurs. Since disengagement is seemingly rather rare, it is hard to get many hints from this experiment, and we might assume that it is reward delivery itself that causes re-engagement. However in a more general setup (e.g. without reward delivery at fixed time points), a mechanism for re-engagement is necessary. One possible way to do that would be by stochastically re-engaging based on either the reward prediction error or expected value. Such a mechanism of re-engagement could happen at any time point but would be extremely likely to happen at the delivery of reward, as well as for the initiation of a new trial. To be fully generalizable we also need to specify the case for disengagement at the time of an action selection. While in a disengaged state we envision the animal not performing an explicit choice, thus potentially not responding within an allocated time. If a choice is required to progress in the behavioral setup it would happen after an eventual re-engagement.

The model raises some further questions. First, we assumed that the probability of disengagement is a function of the actual prediction. However, it is possible that this function scales with the overall magnitude or scale of possible rewards, making the degree of observing relative rather than absolute. There is a report that phasic dopamine itself scales in an adaptive manner [39], , and this would be a natural substrate.

A second issue is whether disengagement is occasioned by the change in predictions associated with the phasic dopamine activity, or the level of the prediction itself. If the former, then in tasks such as the one studied by Bromberg-Martin and Hikosaka [10], where substantial prediction errors only happen with phasic targets and cues, the state could, for instance, just be poorly established in working memory at the outset, because of a weak dopamine signal, and this could lead to a subsequent chance of disengagement. We adopted the simpler scheme in which it is the ongoing predictive value that controls the chance of disengagement. One experiment that hints in the direction of change is that of Spetch et al. [52] (for a more recent study see [53]). In this, pigeons were given the choice between a certain (100%) or uncertain (50%, but observed) reward. Surprisingly, the level of engagement to the latter (measured by the number of pecks to the illuminated key) was many times to that of the former, and the pigeons duly made the suboptimal choice. The model presented in this paper does tie engagement to choice in a similar way, but we would be unable to explain such a strong effect. A variant of the model for which engagement is governed by prediction errors rather than predictions would show some contrast effect that could favor the uncertain, but observed, reward. However, it would be hard to explain such a stark contrast.

A third issue is whether disengagement is complete (and stochastic), or partial (and, at least possibly, deterministic). We considered the former case, and indeed, this leads to a straightforward prediction that the histogram of the dopamine response at the time of a delivered reward in the non-discriminative case might have two peaks; one associated with continuing engagement to the point of reward; the other, which would be roughly twice as high, associated with prior disengagement. However, it is also possible that less dramatic changes in engagement occur during the interval between cues and reward. If many individual neural elements are involved in the engagement (for instance in working memory circuits devoted to timing), then some could disengage before others. This might even lead to a non-uniform behavior among different dopamine cells. Unfortunately, the low firing rates of these cells make it hard to discriminate between these various possibilities.

Finally, the question arises as to the computational rationale for value-dependent disengagement. Other instances of Pavlovian misbehavior, such as withdrawal from cues associated with predictions of low values, can find plausible justifications in terms of evolutionary optimality. Disengagement might be seen in the same way, as a Pavlovian spur to exploration [54] in the face of poor expected returns.

From the perspective of conditioned reinforcement, our account suggests that the issue that is often studied is not really the one that is critical. Various investigators (see, for instance, the ample discussion in Lieberman et al. 1997 [55] about the differences between their findings and those of Fantino and Case 1983 [56]) have considered whether stimuli like S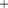 are conditioned reinforcers because of their association with the reward. For us, S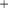 and S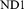 and S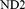 are all conditioned reinforcers. The key question for observing behavior is instead an apparent concavity: the average worth of two different stimuli associated deterministically with small and large rewards is greater than the worth of a single stimulus associated stochastically with the same outcome statistics (see [57]). It is this non-linearity that demands explanation, and not merely the fact, for instance, of savoring or anticipation of the future reward, which could quite reasonably also be purely linear. Some accounts put the weight of the non-linearity onto the stimulus associated surely with the large reward. By comparison, our account places this emphasis onto the non-discriminative stimuli, suggesting that they are more likely to lead to disengagement. The same is true of other sources of non-linearity, for instance a mechanism that accumulates distress from the prolonged variance/uncertainty in the non-discriminative pathway.

Various versions of the ‘observing task’ have also been tested on humans [55], [56], [58]. These studies have shown consistent observing behavior, but, partly because of the different reading of the issue of conditioned reinforcement to the one discussed above, have often focused on different questions and methods from those in Bromberg-Martin and Hikosaka [10]. For instance, one question has been whether subjects would observe if they only ever found out S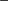 and never S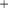 – the idea being that conditioned reinforcement could support observing of the latter but not the former. Unfortunately, the answers have been confusing [55], perhaps partly because of issues about how cognitive effects (e.g., expectations of controllability) influence the results. Note, in particular, that we have only modeled observing behavior associated with repeated experience and learning, and not the sort of single-instance decisions that are often used in human cases.

In conclusion we have shown that the often observed effect of ‘observing’, preferring a behaviorally irrelevant discriminating stimulus cue, can readily be explained by a bias caused by Pavlovian misbehavior, putting it in the same category as a range of other suboptimalities. Informational accounts, however seductive, are not necessary.

## Methods

We model value learning using a modified version of a standard temporal difference model [21], [22]. We assume the task can be specified as a Markov process, where the participant estimates the expected long run future reward (value) of each state 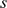 as 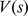, updating it according to
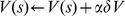
(1)where 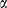 is the learning rate, and 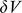 is the change in expected value given by:
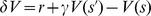
(2)where 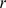 is the delivered reward, and 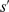 is the state that follows 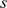. Learning proceeds for all three sorts of trials (forced disc., forced non-disc. and choice trials). The modelled dopamine signal for Figures 4 and 5 is 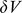.

The only deviation from the standard TD model is in assuming that the correct updating of this system is dependent on maintaining engagement, for instance in working memory. We assume the probability of disengagement of the course of state 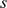 to be
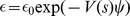
(3)per unit of time (in seconds). Hence, for a given state 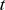 the probability of a correct updating is given by 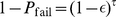, where 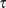 is the amount of time spent in the state (see Figure 1). 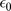 and 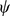 are fixed parameters. We assume the consequence of disengagement to be the transition to a specific fixed (non-updating) state 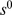 of value 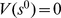 and hence the updating signal for 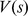 is
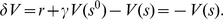
(4)


The system stays in this state, until a reward is delivered at the end of the trial. At this point the system is ‘re-engaged’ creating a TD error relative to the fixed state 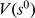 (see Figure 5). We assume that any potential disengagement in the intertrial interval is negated by the initiation of a new trial.

Choice is only possible at one state 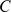, between progressing to either state 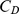 and state 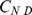. Given the learned values, we assume the subject performs choice 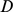 based on the Softmax or Luce choice rule [59]

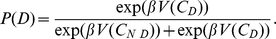
(5)


Note that it is straightforward to see that this version of softmax is dependent on the difference in values (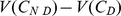), whereas using the logarithm of the value (as in Figure 7c) causes the function to be dependent on the ratio of values (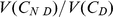).

In the limit without any failures in updating the learned values would approach the true value 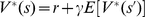, where the expectation is taken over states 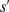. However with a chance of failure 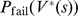 dependent on the value, the iterative solution in Figure 7c can be given by solving
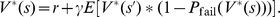
(6)numerically.

For all figures we assumed 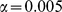 and 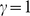. For Figs. 2 and 6 we used parameters, 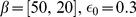 and 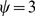. For the aversive stimuli in Figure 7a–b we assumed negative reward values. For Figure 7a the parameters were 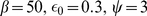. For Figure 7b the parameters were 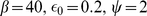. For Figure 7d the parameters were 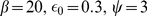. To mimic the fact that dopamine neurons have less dynamic range for increases than decreases in firing rate, for Figure 4 we truncated the negative responses at −25 percent of the maximal positive response of the neuron.
